# P36 Using the Australian National Antimicrobial Prescribing Survey portal (NAPS) to benchmark antibiotic prescribing within an Integrated Care Board (ICB): a pilot point prevalence survey (PPS) of two NHS Trusts

**DOI:** 10.1093/jacamr/dlad066.040

**Published:** 2023-06-14

**Authors:** Nicola Jones, Bee Yean Ng, Bernadette O’Riordan, Aarash Ahmadi, Jean O’Driscoll, Claire Brandish

**Affiliations:** Oxford University Hospitals NHS Trust (OUHFT), UK; Oxford University Hospitals NHS Trust (OUHFT), UK; Oxford University Hospitals NHS Trust (OUHFT), UK; Buckinghamshire Healthcare NHS Trust (BHT), UK; Buckinghamshire Healthcare NHS Trust (BHT), UK; Buckinghamshire Healthcare NHS Trust (BHT), UK

## Abstract

**Background:**

PPS is useful in evaluating volume and appropriateness of antimicrobial prescribing to guide specific antimicrobial stewardship (AMS) interventions. Integrated care systems enable collaboration within an ICB and benchmarking of antimicrobial prescribing in constituent trusts to identify disparities and learn from sharing best practice. NAPS is a web-based qualitative auditing platform which is used to conduct an annual Australia-wide PPS. To assess the suitability of NAPS in benchmarking antimicrobial prescribing practices, we piloted the NAPS platform in the acute admission ward areas in two NHS Trusts within an ICB.

**Methods:**

In OUHFT, admissions to 2 acute medicine wards on a single day were extracted using the Cerner electronic prescribing system. Antimicrobial prescriptions were anonymized, reviewed using NAPS protocol and uploaded to the NAPS platform. In BHT, antimicrobial prescriptions on 2 admission wards were collected manually using the NAPS parameters and audit standards and entered directly into Excel^®^. Ethical approval was not required due to the anonymized nature of this audit.

**Results:**

A total of 124 patients at OUHFT and 53 patients at BHT were included. The proportion of patients prescribed antimicrobials varied, with a prevalence of 23% (*n* = 29) at OUHFT and 54% (*n* = 28) at BHT. The total number of antimicrobial prescriptions was approximately 40 per trust. Compliance with guidelines for OUHFT and BHT (88% versus 83%) and stewardship appropriateness (73% versus 80%) were similar. The main indication for antimicrobials was pneumonia. The most common reasons for inappropriateness were ‘incorrect duration’ and ‘spectrum too broad’. The most prescribed antimicrobials differed, with co-amoxiclav and amoxicillin (43%) at OUHFT and metronidazole and clarithromycin (24%) at BHT. Non-compliance with guidelines was frequently associated with the use of doxycycline and co-amoxiclav (47%) at OUHFT and amoxicillin and piperacillin/tazobactam (72%) at BHT.

**Conclusions:**

Benchmarking of antimicrobial prescribing was possible as both PPSs were conducted using the NAPS parameters and standards. Focusing efforts on antibiotic prescribing for pneumonia, the use of broad-spectrum antimicrobials and reducing duration of courses will be taken forward as a key focus for AMS efforts. NAPS is a suitable tool for benchmarking, sharing best practice and facilitating discussion of AMS interventions across an ICB. This model should be considered as a template for national benchmarking of antimicrobial prescribing.

